# Haplotype-Resolved Assembly in Polyploid Plants: Methods, Challenges, and Implications for Evolutionary and Breeding Research

**DOI:** 10.3390/genes16060636

**Published:** 2025-05-27

**Authors:** Zhenning Zhao, Tao Shi

**Affiliations:** 1 State Key Laboratory of Plant Diversity and Specialty Crops, Wuhan Botanical Garden, Chinese Academy of Sciences, Wuhan 430074, China; zhaozhenning24@mails.ucas.ac.cn; 2 Hubei Key Laboratory of Wetland Evolution & Ecological Restoration, Wuhan Botanical Garden, Chinese Academy of Sciences, Wuhan 430074, China; 3 University of Chinese Academy of Sciences, Beijing 101408, China

**Keywords:** polyploidy, evolution, crops, genome assembly

## Abstract

Polyploidization has been one of the key drivers of plant evolution, profoundly influencing plant adaptation in nature and crop traits in agriculture. Deciphering polyploid genomes is a crucial step for understanding evolutionary history and advancing agricultural applications. However, the inherent complexity of polyploid genomes has long hindered accurate assembly and annotation. Recent advances in sequencing technologies and improved assembly algorithms have significantly enhanced the resolution of complex polyploid genomes. These innovations have led to the successful assembly and public release of an increasing number of high-quality polyploid plant genomes. This review summarizes the mechanisms of polyploid formation and their evolutionary relevance, with a focus on recent technological progress in sequencing and genome assembly. On this basis, we further discuss the current key challenges of polyploid genome assembly and the ways to address them.

## 1. Introduction

Polyploidization events constitute a pervasive evolutionary mechanism in plant macroevolution and have been considered as one of the major drivers of their diversification [1,2]. Empirical studies suggest that approximately 25–30% of angiosperms are polyploids [3]. Polyploidy also plays a crucial role in plant survival, as polyploid plants often exhibit greater environmental adaptability and stress resistance compared to their diploid counterparts in many circumstances [4,5,6]. Furthermore, polyploid plants play a crucial role in agricultural production. They are widely distributed among modern crops and have made significant contributions to crop breeding. Economically significant crops, such as wheat, cotton, potato, sugarcane, and oat are polyploids [7,8,9,10,11]. Despite the scientific and economic value of polyploid plants, their genomic complexity, which often far exceeds that of diploids, has posed considerable challenges to genomic research. For a prolonged period, the study of polyploid genomes remained lagging behind that of diploids. However, in recent years, the integration of third-generation sequencing (TGS) technologies with advanced genome assembly algorithms has progressively enabled the haplotype-resolved assembly and in-depth analysis of most polyploid plant genomes [12].

## 2. The Formation Mechanisms of Polyploid Plants

Polyploid plants are widely distributed in nature, a study by Mayrose et al. involving approximately 2500 vascular plant species revealed that about 33% of them are polyploids [13]. Research indicates that polyploid formation predominantly occurs through three primary mechanisms: the fusion of unreduced gametes, somatic chromosome doubling, and polyspermy [14,15]. The first two mechanisms are commonly observed in plants [16,17], whereas polyspermy has been documented only in certain Orchidaceae species [18]. Polyploids can be categorized into two types based on their formation mechanisms: autopolyploids and allopolyploids. Autopolyploids arise from whole-genome duplication (WGD), leading to chromosome doubling or multiplication within a single species. As a result, they retain high levels of homologous chromosome pairing and genetic redundancy, which can influence genome evolution, gene expression regulation, and adaptation. Autopolyploids can be traced back to a single ancestral species, distinguishing them from allopolyploids, which result from hybridization between different species, followed by genome doubling/multiplication, incorporating genetic material from two or multiple ancestral species [19,20]. Both autopolyploids and allopolyploids are widely distributed in nature. Representative autopolyploid species include wild sugarcane (*Saccharum spontaneum*) and alfalfa (*Medicago sativa*), while typical allopolyploid species include common wheat *(Triticum aestivum*) and cultivated strawberry (*Fragaria* × *ananassa*).

The gradual return to a diploid following WGD is a common phenomenon in nature. If the ancestral species of an organism underwent a WGD followed by rediploidization, resulting in a return to a diploid, the organism is referred to as a paleopolyploid. In contrast, species that have not yet undergone rediploidization and still retain the polyploid genome are termed neopolyploids. For instance, crops such as soybean (*Glycine max*), maize (*Zea mays*), and rice (*Oryza sativa*) have all undergone at least one ancient WGD in their evolutionary history [21,22,23]. Paleopolyploidy is thought to enhance plant survival during environmental upheavals, including the K-pg mass extinction event, and drive the diversification of extant angiosperm lineages [3,24,25].

In this article, we mainly discuss the neopolyploid plants. For the neopolyploid plants, the changes in gene regulation during the process of polyploidization in plants are highly complex [26]. To investigate the complex gene regulatory mechanisms associated with polyploidization, Prost et al. generated a triploid *Chlamydomonas* strain (algae) and subjected it to 425 generations of cultivation. The results revealed nonadditive gene expression patterns widespread in *Chlamydomonas*, with a predominant bias toward the haploid parent. Over the course of the experiment (after 425 generations), the genome size was reduced by 22.3%. In addition, disruptions in protein homeostasis were observed, which gradually recovered during evolution. This recovery may represent a key mechanism enabling rapid adaptation following genome duplication and merging [27].

## 3. Polyploidy-Driven Adaptation and Domestication in Wild and Cultivated Plants

Polyploidy is frequently observed in wild plant species as a consequence of genome duplication. Genome duplication can indeed provide genetic redundancy, potentially masking deleterious recessive mutations through allelic complementation and promoting ecological adaptability. For example, natural autopolyploid populations of *Arabidopsis arenosa* have been reported to exhibit greater tolerance to high-altitude environments, as evidenced by their expanded ecological range compared to diploid counterparts [28]. Similarly, the recurrent formation of allopolyploid *Tragopogon miscellus* within a few generations post-hybridization highlights polyploidy’s potential to drive rapid speciation [29]. Yet, the long-term stability of these neopolyploids, particularly their persistence in natural populations and susceptibility to genomic rearrangements, remains poorly characterized [30]. While polyploidy can act as a catalyst for evolution, its outcomes are sometimes unpredictable. The performance of polyploid plants is not universally beneficial. Some polyploid species may represent evolutionary dead ends, as seen in cases where they grow less well than diploids in stable environments [15]. These complexities highlight the need for integrative research that combines genomic, ecological, and evolutionary perspectives to better understand the adaptive potential and long-term consequences of polyploidy.

In contrast, polyploidy in domesticated plants is typically a result of deliberate selection. Depending on the chosen direction, polyploid crops often exhibit heterosis, enhanced biomass, seedlessness, and increased tolerance to biotic and abiotic stress. For instance, tetraploid cotton (*Gossypium hirsutum*) and common wheat (*T. aestivum*) owe much of their yield potential to subgenome complementation and gene dosage effects. In terms of biotic stress resistance, tetraploid rice and potato exhibit enhanced resistance to *Magnaporthe oryzae* and *Phytophthora infestans*, respectively [31,32].

While polyploid crops offer significant agricultural advantages, artificially induced polyploidization poses biological challenges requiring deeper investigation. For instance, polyploidy may destabilize gene regulatory networks, induce epigenetic fluctuations, and impair chromosomal synapsis during meiosis. Consequently, when exploiting polyploidy for crop enhancement, continuous tracking of transcriptional reprogramming and systematic evaluation of molecular consequences—particularly chromatin reorganization and allele-specific expression patterns—should supersede exclusive reliance on macroscopic trait modifications.

## 4. Advances in Genome Sequencing Technologies: Lessons from the *Arabidopsis* Genome

**Figure 1:**
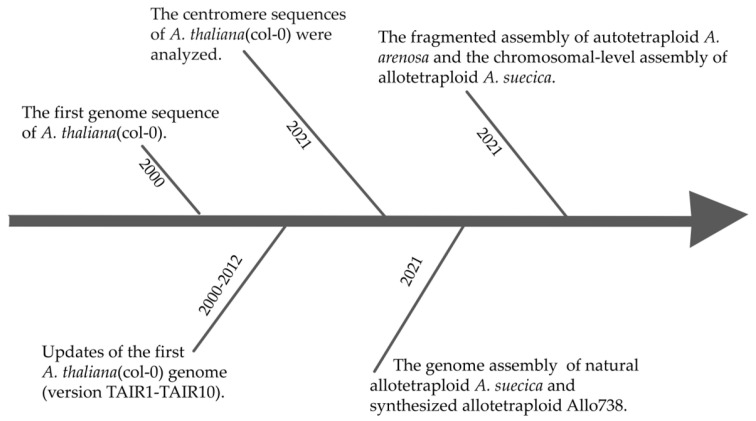
Timeline of *Arabidopsis* (col−0 and polyploids) genome assembly advancements.

Genome assembly is a crucial first step in studying polyploid genomes and depends on continuous improvements in sequencing technologies. Based on first-generation sequencing (Sanger sequencing), the *Arabidopsis* Genome Initiative published the genome of *A. thaliana* (the model plant) in 2000 and it has since undergone multiple revisions (TAIR1-TAIR10) (Figure 1) [33,34]. This version of the genome contains numerous gaps, primarily located in highly repetitive regions such as centromeres and telomeres. Despite the high accuracy of Sanger sequencing, its low throughput and high cost have significantly limited its application. To address the limitations of high cost and low throughput associated with Sanger sequencing. Next-generation sequencing (NGS) technologies based on massively parallel sequencing were developed. NGS technologies significantly reduce the cost of genome sequencing but a major limitation of NGS is its short read length, which poses challenges for assembling complex genomes, particularly those with high levels of repetitive sequences. This issue was not effectively addressed until the advent of third-generation single-molecule sequencing technologies. Oxford Nanopore sequencing and PacBio sequencing have simultaneously addressed the challenges of read length and throughput in sequencing [35,36,37]. Nanopore sequencing achieves an N50 read length of over 100 kb but with a low sequencing accuracy [38], while PacBio sequencing offers read lengths exceeding 20 kb with a high sequencing accuracy of 99.99% [39]. The emergence and advancement of third-generation sequencing (TGS) technologies have significantly improved the decoding of highly repetitive regions such as centromeres and telomeres in plant genomes. With the help of TGS, a nearly complete assembly of *A. arenosa* was achieved in 2021. Around the same time, several polyploid *Arabidopsis* species were reported; however, the autotetraploid *A. arenosa* genome remains fragmented due to its high allelic similarity (Figure 1) [28,33,40,41]. This discrepancy highlights a persistent technical challenge: current sequencing, though powerful, is still not fully equipped to resolve complex polyploid genomes, particularly when subgenomic divergence is minimal, such as the autopolyploids; future research needs to focus on developing tailored computational strategies for polyploidy based on the advanced sequencing technology that can disentangle the intricacies of polyploid genomic architecture.

## 5. Advancements in Genome (Contig) Assembly Algorithms

**Figure 2:**
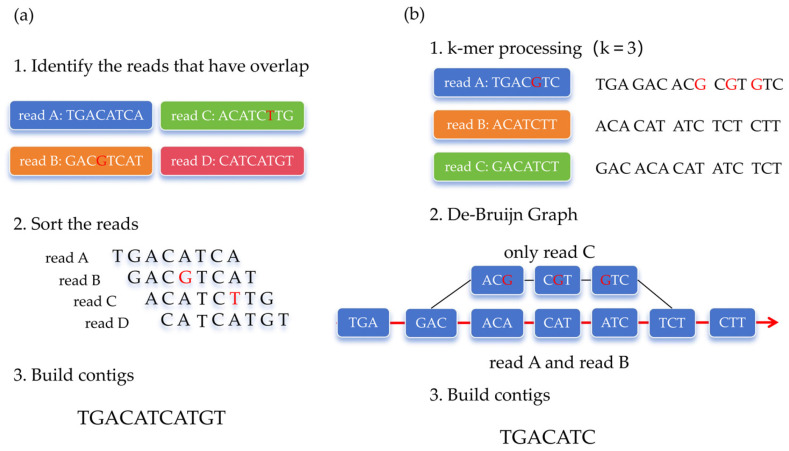
Genome assembly procedure of Overlap Layout Consensus (OLC) algorithm and de Bruijn graph (DBG); sequencing error bases are represented by red. (**a**) Overview of OLC algorithm. (**b**) Overview of de Bruijn graph algorithm. The main assembly path is represented by red arrow.

While the advancement of sequencing technology contributes to better genome assembly, genome assembly algorithms also play a crucial role in improving assembly quality. Currently, three main categories of traditional genome assembly algorithms are commonly employed: greedy algorithms, overlap-layout-consensus (OLC) algorithms and de Bruijn graph. The greedy algorithm typically selects the sequences with high quality, moderate length, and strong uniqueness as seed sequences and then extends seed sequences by iteratively searching for reads that overlap with both ends of the current sequence until no further extension is possible. However, when one seed sequence overlaps with multiple reads, the greedy algorithm struggles to determine the optimal sequence for extension [42]. As a result, the contigs assembled using this approach are often relatively short. Due to these limitations, the greedy algorithm is now rarely used in genome assembly. The overlap-layout-consensus (OLC) algorithm identifies overlaps between reads through pairwise comparisons and constructs sequence paths accordingly. The optimal path is then determined, yielding the corresponding contigs (Figure 2a). OLC algorithms are particularly well suited for long-read sequencing data and their performance degrades significantly when sequencing data are highly fragmented. Due to this limitation, OLC algorithms are primarily applied in the assembly of Sanger and TGS sequencing data [43].

The de Bruijn graph algorithm first fragments the sequencing reads into k-mers of length k with a step size of 1. It then constructs a de Bruijn graph based on the overlap relationships between these k-mers. By identifying a Eulerian path within the graph, the algorithm ultimately assembles the sequence into contigs (Figure 2b). Compared to the OLC algorithm, de Bruijn graph-based assembly does not rely on pairwise read overlap alignments, which significantly reduces memory consumption and improves computational efficiency [44].

## 6. Current Challenges and Strategies in Polyploid Plant Genome Assembly

Assembling polyploid genomes poses significant challenges due to the difficulty in distinguishing highly similar homologous chromosomes, resolving allelic diversity among duplicated genes, high heterozygosity, and managing extensive repetitive sequences, which collectively complicate accurate genome reconstruction and phasing. Plant genomes often contain highly complex regions, such as centromeres, pericentromeres, and telomeres, which are rich in tandem repeats. In recent years, the development of various assembly tools has significantly advanced genomic research. Among them, Canu, NextDenovo, and HiFiasm have demonstrated robust performance in assembling high-quality genomes [45,46,47]. Notably, HiFiasm incorporates high-throughput chromosome conformation capture (Hi-C) sequencing data to enable haplotype-aware genome assembly, facilitating the direct phasing of genomic sequences during the assembly process. This approach enhances the reconstruction of high-contiguity, haplotype-resolved genomes, making it particularly advantageous for the assembly of polyploid and structurally complex genomes [47]. Specifically, HiFiasm first performs error correction on PacBio HiFi reads and then constructs an assembly graph using the corrected data. In this graph, unitigs (non-branching paths) serve as the nodes, and edges represent overlaps between them. A 31-mer index is built for the unitigs in the assembly graph, and Hi-C reads are mapped to these k-mers to identify pairs of distant heterozygous unitigs bridged by Hi-C read pairs. Haplotype-specific links are then added between these unitigs, providing long-range phasing information. Next, a bipartitioning of unitigs is conducted, where the bipartitioning problem is formulated as a graph maximum cut (Max-Cut) problem. A stochastic algorithm is applied to find a near-optimal solution that ensures unitigs within the same partition exhibit low redundancy and share numerous Hi-C links. Finally, unitigs from each partition are concatenated to generate contigs for individual haplotypes. This algorithm innovatively integrates Hi-C data into the contig assembly process to assist in haplotype phasing.

Compared to trio-binning methods, haplotype-resolved assembly based on Hi-C data reduces the dependence on parental sequencing data, thereby broadening its applicability to a wider range of samples. However, the phasing accuracy achieved through Hi-C data is subject to substantial variability. Specifically, Hi-C phasing typically results in a higher incidence of switch errors compared to trio-binning methods. This performance gap is influenced by factors such as Hi-C coverage depth, genomic complexity, and the level of heterozygosity. Furthermore, the algorithm’s ability in polyploid genomes remains limited, especially when contrasted with its performance on diploid species. In summary, while the approach represents a notable methodological innovation, it still demands further methodological refinement and optimization to fully realize its potential in diverse genomes.

**Figure 3:**
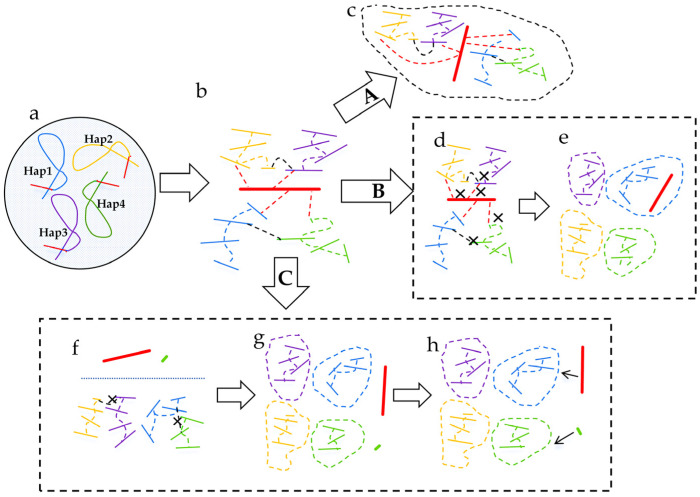
Three strategies for chromosome clustering in polyploid genome assembly. Arrows A, B, and C represent the traditional method, the ALLHiC method, and the HapHiC method, respectively. (**a**) Homologous chromosomes in a polyploid genome, where red regions indicate highly similar homologous segments. (**b**) Hi-C links among overlapping contigs: red lines represent collapse contigs; black dashed lines indicate allele Hi-C links; red dashed lines indicate Hi-C links between collapse and uncollapse contigs. (**c**) Traditional chromosome anchoring approach. (**d**) Hi-C link pruning based on allele tables from closely related species, retaining only the strongest Hi-C signals between collapse and uncollapse contigs. (**e**) Clustering after pruning. (**f**) Preprocessing of contigs, including the removal of low-information sequences, collapse contigs, and inter-allele Hi-C links. (**g**) Markov clustering on processed contigs. (**h**) Reassignment of remaining contigs.

Critically, the presence of multiple sets of chromosomes in polyploid plant nuclei poses significant challenges for genome assembly. In autopolyploids and certain allopolyploids with highly similar subgenomes, the high sequence similarity between haplotypes makes it difficult for current assembly algorithms to accurately reconstruct all haplotypes. This challenge leads to ‘contig collapse’ and ‘chimeric assembly’, resulting in the loss of haplotype-specific sequences and compromising genome completeness and accuracy [48]. To achieve chromosome-scale genome assembly, auxiliary methods are typically required to anchor and order the assembled contigs. Currently, available approaches include genetic maps, optical maps, and Hi-C sequencing [49,50]. Among these, Hi-C is the most widely adopted method. Compared to genetic maps, Hi-C does not require the mapping population and can be implemented using sequencing data from a single individual, making it a more practical and cost-effective solution for chromosome-level scaffolding. LACHESIS was the first software to utilize Hi-C for chromosome scaffolding [51]. Subsequently, tools such as 3D-DNA, SALSA, and YaHS were developed, all demonstrating strong scaffolding capabilities [52,53,54]. However, these tools were originally designed for genome assembly in diploid species. In autopolyploids and allopolyploids with highly similar subgenomes, genome assembly frequently encounters challenges such as contig collapse and chimeric contigs. These issues result in erroneous interaction signals, which interfere with the anchoring and ordering of contigs, ultimately reducing the accuracy and quality of chromosome-level scaffolding. To address the challenges in chromosome scaffolding for autopolyploid genomes, Zhang et al. (2019) developed ALLHiC, a tool specifically designed for polyploid genome assembly [55]. ALLHiC utilizes allele tables constructed from closely related species to filter out Hi-C signal noise caused by high sequence similarity among chromosomes in polyploid genomes (Figure 3). By reducing erroneous pairings, ALLHiC improves scaffolding quality and was successfully applied to assemble several chromosome-scale reference genomes of polyploid *S. spontaneum* [55,56,57].

However, many polyploid species lack suitable closely related reference genomes, which limits the applicability of ALLHiC. To overcome this limitation, Zeng et al. (2024) [58] developed HapHiC, a reference-free Hi-C scaffolding tool. HapHiC introduces a novel algorithm that integrates multiple lines of evidence to identify potential collapsed and chimeric regions, such as Hi-C link density, sequencing depth, and neighborhood density based on rank-sum value. Then, it applies the Markov Cluster Algorithm (MCL) to cluster contigs into groups. After reassigning misplaced contigs, it finally anchors and orders the contigs. This series of computational strategies significantly enhances the accuracy and efficiency of polyploid genome scaffolding, offering a robust solution for polyploid genome assembly [58] (Figure 3). A key innovation of HapHiC lies in the development of a reference-free algorithm for identifying collapsed regions. This suite of methods in HapHiC is particularly suited for the assembly of the polyploid genomes in which subgenomes exhibit high sequence similarity that often leads to collapsed assembly.

In traditional genome assembly pipelines, contig assembly and Hi-C chromosome anchoring are typically disconnected. The information generated during contig assembly often cannot be effectively utilized in the scaffolding process, which hampers the overall efficiency and accuracy of genome assembly. HapHiC addresses this limitation by actively integrating the assembly graph generated by HiFiasm into the chromosome anchoring process and introduces a flexible reference-weighting scheme tailored for this integration. This algorithmic innovation enhances the continuity of the assembly workflow and demonstrates significant potential for further development and application.

Currently, deep learning techniques have also been applied to genome assembly. Jiang et al. (2024) [59] developed AutoHiC, a tool designed to leverage deep learning for comprehensive Hi-C data analysis, enabling efficient and automated error detection and correction. This approach helps to minimize human-induced errors during scaffolding, thereby improving the accuracy and reliability of chromosome assembly. While this approach may help mitigate human-induced biases, its effectiveness is still contingent on model generalizability and the quality of training data. Furthermore, the use of deep learning in genome assembly remains in its early stages, and interpretability, computational cost, and robustness across diverse genomes remain challenges.

With the continuous advancement of assembly technologies, an increasing number of tools are expected to be developed and applied to genome assembly, particularly for the complex task of assembling polyploid genomes. Table 1 lists representative tools employed in polyploid genome assembly.

## 7. Genomic Assembly Studies of Polyploid Plants

In recent years, advancements in genome sequencing technologies and assembly algorithms have significantly accelerated the assembly and publication of high-quality polyploid plant genomes. Constructing a high-contiguity and accurately assembled reference genome is a foundational and essential step in genomic research; here, we summarize some of the genomes of polyploid plant species published in recent years (Table 2). They can be classified into autopolyploids and allopolyploids, encompassing a range of ploidy levels from triploid to nonaploid and most of them possess high economic value. These genomic resources have helped reveal evolutionary histories of many species [56,61,62,63,64,65,66,67,68,69,70,71,72,73,74,75].

The assembly of polyploid plant genomes has greatly facilitated research on the origins of subgenomes in many species. For instance, Li et al. successfully assembled the genomes of two representative triploid banana cultivars, *M. acuminata* cv. Cavendish and *M. acuminata* cv. Gros Michel, and determined that the A subgenome of these two major triploid banana varieties primarily originated from three subspecies of *M. acuminata*: *M. acuminata* ssp. banksii (Ban), *M. acuminata* ssp. malaccensis (Dh), and *M. acuminata* ssp. zebrina (Ze) [61]. Unlike the origin of banana, *B. odashimae* originated from the intergeneric allopolyploidization events. Specifically, *B. odashimae* is an allononaploid, containing three subgenomes derived from *Dendrocalamus* and *Bambusa*, both of which are hexaploid; *Bambusa* contributed two haploid subgenomes, while *Dendrocalamus* contributed only one [75].

In addition, many polyploid species have experienced extensive gene introgression during their evolutionary history, and genomic analyses of these species have provided valuable insights into this process. Bao et al. utilized HiFiasm to assemble a haplotype-resolved genome of tetraploid *S. tuberosum* C88 and found that extensive introgression between cultivated tetraploid potatoes and their wild relatives [63]. Similar to the situation with cultivated potato, genome introgression also influenced chrysanthemum deeply. Song et al. conducted an in-depth study of the *C. morifolium* genome and revealed that *C. morifolium* may have undergone extensive gene introgression during its evolution, suggesting that its genome was likely shaped by widespread introgression between *C. indicum* and *C. nankingense* [73].

Moreover, many previously unresolved questions have been clarified with the aid of genomic data. For instance, whether *A. arguta* is an autotetraploid or allotetraploid has been a long-standing debate. Lu et al. confirmed that *A. arguta* is an autotetraploid by genome assembly. Phylogenetic analysis further indicated that its tetraploidization event occurred approximately 3.13 Mya through genome doubling from a diploid ancestor [64].

Although genome assembly and analysis have advanced our understanding of polyploid species, the evolutionary histories of certain plant taxa remain unresolved, largely due to the intricate and obscure origins of their subgenomes. In 2019, Edger et al. successfully assembled the first genome of cultivated strawberry (*F*. × *ananassa*, 2*n* = 8*x* = 56) and hypothesized that its four subgenomes originated from four distinct diploid ancestors: *F. vesca*, *F. iinumae*, *F. viridis*, and *F. nipponica* [76]. However, further studies only considered that *F. vesca* and *F. iinumae* are extant progenitor species of cultivated strawberry [77,78]. These conflicting interpretations highlight the limitations of current genomic evidence in fully analyzing polyploid ancestry. It is difficult to analyze the origins of complex species solely relying on genomic data of limited taxonomic samples. Future research requires more integrative approaches that combine comparative genomics, phylogenetics, and cytological data for a comprehensive analysis of polyploid species to resolve the evolutionary histories of the complex species.

## 8. Future Development and Prospects

A high-quality reference genome serves as the cornerstone of genomic research. Now, the genomes of many polyploid plants have been successfully sequenced and assembled, providing critical resources for understanding polyploid evolution, genome organization, and gene expression dynamics. Despite these advancements, accurate genome assembly remains a formidable challenge, particularly in resolving highly complex centromeric and pericentromeric regions, transposable elements, and tandem repeats. While some polyploid plant species have now achieved telomere-to-telomere (T2T) genome assemblies [79], high-quality reference genomes are still lacking for numerous polyploid species, particularly autopolyploids. With continued improvements in sequencing technologies and genome assembly algorithms, it is anticipated that these challenges will be gradually overcome, enabling T2T genome assemblies for an increasing number of complex polyploid plant species.

Furthermore, with the advancement of genomics, traditional linear reference genome assemblies are increasingly inadequate to meet the demands of many current studies. In many cases, a single plant reference genome fails to comprehensively capture the genetic variation and diversity within a species. To address this limitation, graph-based pangenomes, constructed through whole-genome alignment, utilize graph structures to represent genomic variations across multiple genomes [80]. These structures integrate insertions, deletions, inversions, and translocations within genomic sequences, effectively preserving genetic variation information and providing a more comprehensive representation of intraspecific genetic diversity. Recent studies also have demonstrated the power and applicability of this approach in various plant species, particularly in complex polyploid plants. In 2024, Jiao et al. [81] de novo assembled chromosome-scale genomes of 17 representative wheat cultivars, capturing major structural variations (SVs) within Chinese wheat varieties. Their study provided valuable insights into wheat genetic diversity and breeding history, offering genomic resources for genetic improvement in wheat. Similarly, Li et al. [82] constructed graph-based pangenomes for both diploid and allotetraploid upland cotton using 50 genome assemblies. Comparative analysis between these genomes identified continuously evolving homologous and highly divergent regions, shedding light on the evolutionary history of cotton genomes and providing a genomic foundation for molecular breeding in cotton. Additionally, super-pangenomes covering higher taxonomic levels have been constructed for species such as grape (*Vitis*), rice (*Oryza*), and watermelon (*Citrullus*) [83,84,85]. With the rapid advancement of graph-based pangenome technology, future research on polyploid plants is expected to benefit significantly, facilitating a deeper understanding of their genomic complexity, evolution, and breeding potential.

## Figures and Tables

**Table 1:** List of representative tools for polyploid genome assembly.

Tools	Step in Assembly	Method Breakthrough	Publishing Date	Reference
Canu	Contig assembly	MinHash Alignment Process solved the problem of low alignment efficiency due to high error rate in long-read assembly	2017	Koren, et al. [45]
SubPhaser	Subgenome partitioning	Partitioning polyploid subgenomes based on k-mer frequency statistics	2022	Jia, et al. [60]
HiFiasm	Contig Assembly	Combining the string graph with the phased assembly graph enables haplotype-resolved genome assembly	2021	Cheng, et al. [47]
ALLHiC	Scaffolding	An allele table was constructed from the genome of a closely related species to assist in the scaffolding process	2019	Zhang et al. [55]
HapHiC	Scaffolding	Chromosome anchoring of polyploid genomes can be achieved without relying on reference genomes	2024	Zeng et al. [58]

**Table 2:** Example polyploid plant genomes published in recent years.

Chromosome Ploidy	Common Name	Species	Assembly Size	Publishing Date	Reference
Autotriploid	Banana	*Musa acuminata* cv. Cavendish	1.48 Gb	2024	Li et al. [61]
		*M*. *acuminata* cv. Gros Michel	1.33 Gb		
Autotetraploid	Alfalfa	*M. sativa*	2.738 Gb	2020	Chen et al. [62]
	Wild sugarcane	*Saccharum spontaneum*	2.761 Gb	2022	Zhang et al. [56]
	Potato	*Solanum tuberosum* C88	3,16 Gb	2022	Bao et al. [63]
	Hardy kiwifruit	*Actinidia arguta*	2.61 Gb	2024	Lu et al. [64]
	Fish mint	*Houttuynia cordata*	2.24 Gb	2024	Yang et al. [65]
	Oil tea tree	*Camellia oleifera*	11.06 Gb	2024	Zhang et al. [66]
Allotetraploid	Horseradish	*Armracia rusticana*	610.05 Mb	2023	Shen et al. [67]
	China rose	*Rosa chinensis*	2.51 Gb	2024	Zhang et al. [68]
Autohexaploid	Wild oat	*Avena sterilis*	10.99 Gb	2024	He et al. [69]
Allohexaploid	Wheat	*Triticum aestivum* Chinese Spring v1.0	14.5 Gb	2018	IWGSC et al. [70]
		*T. aestivum* Chinese Spring v2.1	14.41 Gb	2021	Zhu et al. [71]
		*T. aestivum* Chinese Spring	14.446 Gb	2025	Wang et al. [72]
	Garden Mum	*Chrysanthemum morifolium*	8.15 Gb	2023	Song et al. [73]
Allooctoploid	Strawberry	*Fragaria chiloensis*	1.64 Gb	2023	Jin et al. [74]
		*F. virginiana*	1.54 Gb		
Allononaploid	Bamboo	*Bambusa odashimae*	3.36 Gb	2024	Wang et al. [75]

## Data Availability

No new data were created or analyzed in this study. Data sharing is not applicable to this article.
